# A Machine Learning Approach for Specification of Spinal Cord Injuries Using Fractional Anisotropy Values Obtained from Diffusion Tensor Images

**DOI:** 10.1155/2014/276589

**Published:** 2014-01-21

**Authors:** Bunheang Tay, Jung Keun Hyun, Sejong Oh

**Affiliations:** Department of Nanobiomedical Science, Dankook University, Cheonan 330-714, Republic of Korea

## Abstract

Diffusion Tensor Imaging (DTI) uses in vivo images that describe extracellular structures by measuring the diffusion of water molecules. These images capture axonal movement and orientation using echo-planar imaging and provide critical information for evaluating lesions and structural damage in the central nervous system. This information can be used for prediction of Spinal Cord Injuries (SCIs) and for assessment of patients who are recovering from such injuries. In this paper, we propose a classification scheme for identifying healthy individuals and patients. In the proposed scheme, a dataset is first constructed from DTI images, after which the constructed dataset undergoes feature selection and classification. The experiment results show that the proposed scheme aids in the diagnosis of SCIs.

## 1. Introduction

The Spinal Cord (SC) is a major pathway for motor and sensory signals traveling between the brain and the peripheral nervous system. The SC, along with the brain, comprises the central nervous system. It is tubular in shape and contains white matter (spinal tracks) and gray matter (neuronal cell bodies). When a Spinal Cord Injury (SCI) occurs, the spinal tracks, which convey sensory, motor, and autonomic signals between the brain and organs, are disrupted. An SCI may cause patients to become paralyzed or stop organs from functioning properly. The International Standards for Neurological Classification of Spinal Cord Injury (ISNCSCI) motor and sensory scores enable us to assess SCI patients more precisely. These scores were developed by the American Spinal Injury Association (ASIA) in 1982 and updated several times [1–3]. They are correlated with functional status, and they are essential to arriving at a prognosis for SCI patients in clinical rehabilitation units [4]. However, clinical assessment based on ISNCSCI scores has limitations for accurate diagnosis of SCIs. It includes unclear information when there are concomitant injuries in other organs, and it is also somewhat subjective as it relies on information relayed by the patient.

**Figure 1:**
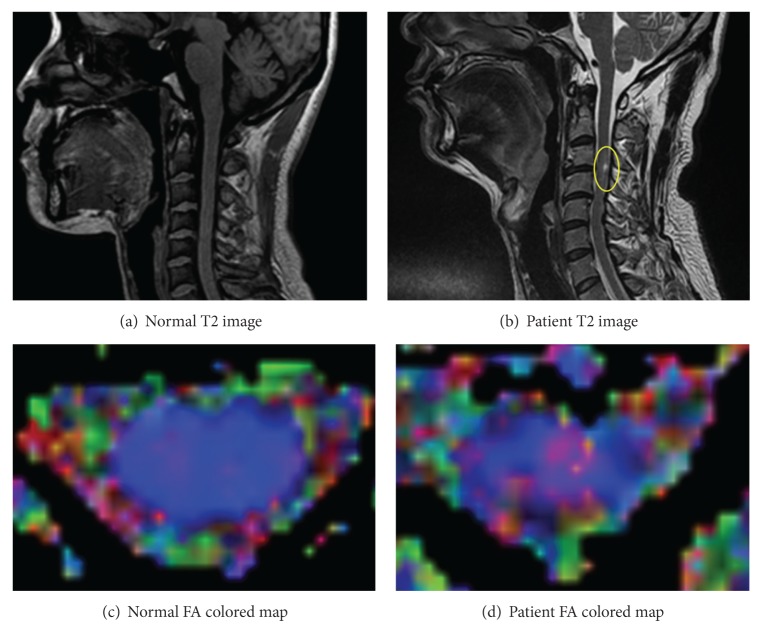
Spinal cord image. In (b), yellow circle shows injured area. In (c) and (d), the color of each pixel means direction of water molecules in SC tissues. In the patient FA map, the direction is irregular because it includes disconnected part of nerves and water molecules cannot flow straight way.

Conventional Magnetic Resonance Imaging (MRI) is widely used to diagnose SCI. MRI is a medical imaging technology that produces high-quality images of organs and tissues at the macroscopic level. It utilizes a black and white contrast image to differentiate between soft and hard tissues. Diffusion Tensor Imaging (DTI) is an advanced technology that utilizes echo-planar images obtained from MRI. It maps the diffusion of water molecules in the brain and SC tissue according to their tissue structure and architecture (Figure 1). DTI is used in the study of diseases and pathological conditions such as multiple sclerosis, brain trauma, brain tumors, and hypertensive encephalopathy. It provides numerical information about the magnitude and orientation of each individual tissue in a three-dimensional (3D) space. This is termed as diffusion anisotropy. The diffusion tensor consists of many diffusion ellipsoids floating in space. The orientation of each diffusion ellipsoid is described via a set of orientation vectors, also known as eigenvectors. When an eigenvector changes either its length or direction, it produces a different result corresponding to its eigenvalue. In DTI, diffusion anisotropy can be expressed as Fractional Anisotropy (FA). FA is sensitive to the number of fibers with directionality within each voxel and is widely used to measure fiber integrity with a range from 0 to 1. The FA value indicates the degree of water diffusion anisotropic motion, with a higher FA value indicating a higher degree, as represented by the following equations:

(1) $$\begin{array}{c} \text{FA}=\sqrt{\frac{3}{2}}\frac{\sqrt{{\left(\lambda_{1}-\bar{\lambda }\right)}^{2}+{\left(\lambda_{2}-\bar{\lambda }\right)}^{2}+{\left(\lambda_{3}-\bar{\lambda }\right)}^{2}}}{\sqrt{\lambda_{1}^{2}+\lambda_{2}^{2}+\lambda_{3}^{2}}},\\ \bar{\lambda }=\frac{\left(\lambda_{1}+\lambda_{2}+\lambda_{3}\right)}{3}. \end{array}$$

In the equations, the corresponding eigenvalues *λ* give the magnitude of the peak in that direction.

In this paper, we propose a novel SC analysis scheme that uses a machine learning technique. Our goal is to provide support for diagnosis of SCI by human experts. At present, human experts analyze DTI images and FA values and then make decisions based on their experience. We believe that if we can provide them with more objective information, they will be able to make more accurate diagnoses. In machine learning, classification schemes are used for prediction or diagnosis. However, training data is required for classification tasks. Therefore, in our scheme, we construct a training dataset using four FA values from DTI slice images taken from patients and normal controls. We then expand the base dataset to a higher dimensional dataset comprising 15 features and abstract the target dataset using a feature selection algorithm to improve classification accuracy. The resulting dataset has a prediction accuracy of more than 90%. Our contributions are (1) a training dataset from the DTI image construction process and (2) application of a classification scheme to the prediction of SCIs. The size of the DTI data associated with an individual is over 200 MB. Raw DTI data is large and difficult to use in computer-aided diagnosis. In our scheme, we extract useful numeric data from the raw DTI data and then use it for diagnosis. Our approach can be applied in any area in which DTI is utilized for diagnosis.

## 2. Related Work

T1- and T2-weighted imaging (Figure 1) have been performed in sagittal and axial panels in order to evaluate SCI individually. The neurological level of injury and the severity were determined by clinical assessments, and conventional MRI was used to detect the level of signal changes within the injured spinal cord. The signal change level was correlated with clinical findings [5]. We used a classification scheme to automatically identify the image slices of injured patients. The scheme provides speedy and accurate results, and it works effortlessly with pattern recognition mechanisms. Classification accuracy is a significant measure of the quality of a prediction scheme. Many researchers have attempted to increase classification accuracy by manipulating the dataset or by improving the classification algorithms. As stated in Section 1, our objective is to abstract FA values from DTI images and thereby aid human experts in ascertaining which part is injured. Even though an FA value is a reliable measure for the specification of SCI, human experts rely heavily on their individual experiences gained from previous analyses of T1- or T2-weighted images. We believe that an automated method that can pinpoint the injured part and provide related information would be invaluable for SCI diagnosis.

Machine learning covers a wide ranging area and mostly uses computation models in a variety of real-world domains. In machine learning, the focus is on the design and development of algorithms that can make decisions as humans do, based on information from a database. It is used in tasks such as recognition, diagnosis, planning, robot control, and prediction. Machine learning can also be used to predict tissue toxicity [6] and to examine neuroimaging data [7]. Both tasks use pattern recognition, which involves the detection of multiple variables of interest. Nowadays, everything we do involves massive amounts of data that must be managed, analyzed, and utilized by researchers. Machine learning can be used to extract important relationships and correlations that may be hidden within large piles of data. It improves the efficiency of systems and the design of machines. In recent years, image analysis has become a major area of application for machine learning in computer-aided diagnosis, medical image analysis, and lesion segmentation.

Classification is a key theme in biological studies, and machine learning plays an important role in the classification process. Machine learning is used to predict unknown sample data among an already known dataset. It can discriminate between two or more contrary objects, group together similar objects, or separate different objects. In the classification process, the specific properties of objects are used to categorize them, and each object is assigned a class label (e.g., “patient,” “normal,” etc.) that describes the particular group to which it belongs. However, classification mainly operates by making predictions based on known sample data that has been learned from training data. In classification, term training and testing are used to predict the unknown data's labels. We train the classifier with known sample data in a training dataset and check its performance by examining the test dataset, which consists of the unknown sample used to predict its class label. K-Nearest Neighbor (KNN) [8] and Support Vector Machine (SVM) [9, 10] are well-known classification algorithms. KNN is an instance-based learning classifier that performs classification based on the closest data point in feature space. The KNN algorithm is outlined in Algorithm 1.

Feature selection preserves classification accuracy and reduces the number of features that are irrelevant and redundant. It is a statistical machine learning technique used to build a strong and stable predictor and is often viewed as a preprocessing step for classification. Feature selection is not only used to handle noisy data but also to cope with very large datasets. Due to its dimensional reduction of the data, feature selection enables a classification algorithm to be faster and more effective. Feature seLection (FeaLect) [11], Feature Selection based on a Distance Discriminant (FSDD) [12], ReliefF [13], Clearness-Based Feature Selection (CBFS) [14], and *R*-value-based Feature Selection (RFS) [15] are well-known feature selection algorithms. RFS is based on the *R*-value [16], which is a measure used to capture the congestion area among classes in a feature. The basis of the FSDD algorithm is to identify the features that result in good class separability between classes and ensure that samples in the same classes are as close as possible. ReliefF is regarded as one of the most successful feature selection algorithms. The basic idea underlying ReliefF is to iteratively estimate feature weights according to their ability to discriminate between neighboring instances. CBFS is a very efficient feature selection algorithm based on “CScore” measures. CScore calculates the degree of samples located in the correct class region. Based on the Lasso, FeaLect introduces an alternative algorithm for feature selection. This algorithm measures the “quality” of each individual feature by defining a scoring scheme. Training data is used to generate several samples, select high relevance-ordering of features for each sample, and finally combine the highly relevant features in each relevance ordering together. In this study, we mainly used feature selection to evaluate the discriminative power of each feature and to find the most accurate feature subset.

## 3. Materials and Methods

**Figure 2:**
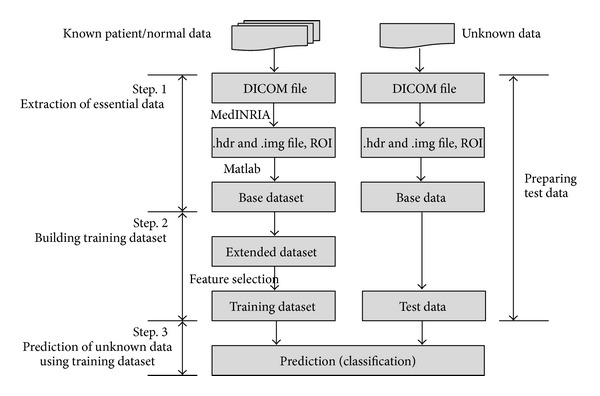
Preview of the classification analysis procedure for the new dataset.

Figure 2 summarizes our proposed approach. The left side of Figure 2 illustrates the training dataset (target dataset) production process, while the right side shows the production of the test data in which it is not known whether the subject is a normal subject or a patient. After building the training dataset, we perform prediction analysis using the test data. The steps used to prepare the test data are the same as those used to build the training dataset. To build the training dataset, we acquired DTI image data from the Dankook University Hospital (Cheonan, Republic of Korea). The SCI was then clinically evaluated by applying ISNCSCI standards [17]. The DTI raw data was in the Digital Imaging and Communications in Medicine (DICOM) format. We extracted FA values from the DICOM file using MedINRIA Ver. 1.8.0 (available free at http://www-sop.inria.fr/asclepios/software/MedINRIA/) and Matlab Ver. 2007a (http://www.mathworks.com/products/matlab/), and we used these values to build a base dataset with four features. We then extended the base dataset and processed the target dataset through feature selection. Finally, we conducted classification analysis.

### 3.1. Extraction of Essential Data

**Figure 3:**
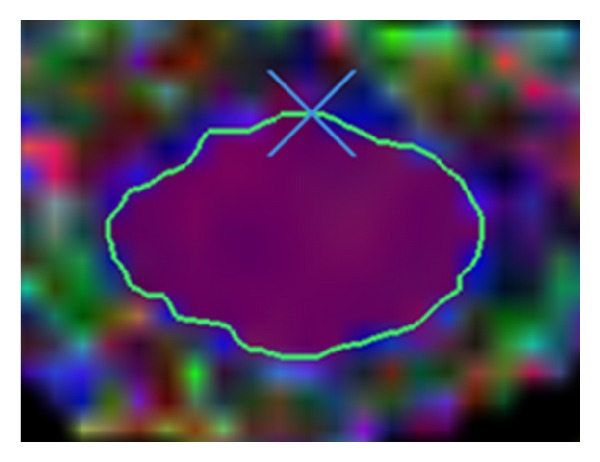
Example of ROI in colored FA.

The clinical data acquired from Dankook University Hospital was from 14 individuals (9 patients and 5 normal controls). The nine patients had SC problems near the neck (see Figure 3). To generate one DTI file, an aggregate image of approximately 26 to 28 slices was captured from the midbrain to SC level T1 or T2. For simplicity, we eliminated the slices above the C1-2 level and below the C7 level that may cause some artifacts. We placed the rest of the slices into one of two categories: “injured” or “normal.” T1 or T2-weighted sagittal images were then used to check the level of those exhibiting abnormal signal intensities within the cervical SC. We matched the axial images with the sagittal section to check which axial slices were abnormal. These axial images, which were obtained with conventional T1 or T2 MRI, were also compared to the axial section in Echo Planar Imaging (EPI). The ANALYZE image (.hdr and  .img files) and Region of Interest (ROI) were generated using the dedicated software MedINRIA. To select the ROI, we paid special attention to the void region of gray matter, blank areas, motion artifacts, and CerebroSpinal Fluid (CSF) partial volume effects. ROIs were defined in every slice in the axial plane (Figure 3).

We took the diffusion tensor measure (FA) from DTI and Fiber Tracking [18]. The gradient table must also be validated with the DTI tool, which describes magnetic field information to render the diffusion image in a particular direction. We imported the ROIs into the Matlab program as a binary mask to filter the SC regions using the Neuroimaging Informatics Technology Initiative (NIfTI) and ANALYZE image tools [19]. The imported ROIs were then rotated by 90° to generate the DTI. The ROIs were maintained at the same coordinates and direction.

**Figure 4:**
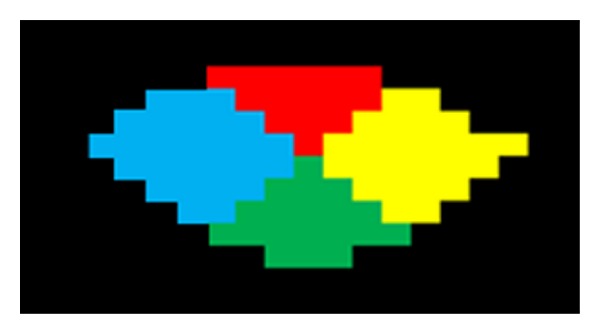
Divided spinal cord regions (red: posterior, green: anterior, blue: left, yellow: right).

After the SC region was filtered, we subdivided the selected SC region into four different subregions: posterior, anterior, left, and right. We then applied the result individually on each axial plane and computed the subdivision as the cumulative intensity along the *X*- and *Y*-axes. The center point on the spine area (ROI as mask) was chosen in both the *X* and *Y* directions. To create four separated segments, we rotated the line joining the *X* and *Y* center points by 45°; the intersection and ROI were considered as bounding lines to separate the regions into anterior, posterior, left, and right subregions (Figure 4) [20]. In the division process, we balanced each voxel in each region on the central point.

**Figure 5:**
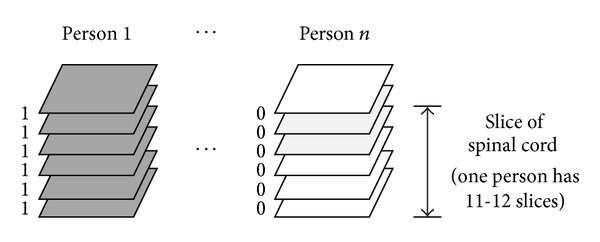
Class labeling for each slice image (gray: injured slice, white: normal slice).

For the features of the base dataset, we derived the mean value of each region in every slice. This is a simple way to acquire SC information from the FA value; it is clear, fast, and distinguishable between normal and abnormal slices. In the final step, we had four features of each slice that described the condition of the SC through numerical data. To prepare for classification analysis, we labeled each slice, which had been examined by experts at Dankook University Hospital, as either class 0 (patient) or class 1 (normal control) (Figure 5). This provided our base dataset.  Table 1 presents an example of a base dataset. Our dataset had 164 data samples comprising 106 injured and 58 normal slices.

### 3.2. Building the Target Training Dataset

In this section, we outline the method used to expand the base dataset to a target training dataset in order to obtain high classification accuracy. This approach is useful due to the maximization of the base dataset potential. We have the original four features: posterior (*P*), anterior (*A*), left (*L*), and right (*R*). Hence, the feature set *S* ^*B*^ of the base dataset is

(2) $$\begin{array}{c} S^{B}=\left\{P,A,L,R\right\}. \end{array}$$

Based on *S* ^*B*^, we expand the base dataset to feature set *S* ^*T*^ of the target dataset by taking all combinations of the four features. *S* ^*T*^ is as follows:

(3) ST={P,A,L,R,(P,A),(P,L),(P,R),(A,L),(A,R), (L,R),(P,A,L),(A,L,R),(P,A,R), (P,L,R),(P,A,L,R)}.

The feature value of (*P*, *A*) is the average of *P* and *A*, and the feature value of (*P*, *A*, *L*) is the average of *P*, *A*, and *L*. The total number of combinations from *n* features is 2^*n*^ − 1. Therefore, the number of features in *S* ^*T*^ is given by

(4) $$\begin{array}{c} n\left(S^{T}\right)=2^{n}-1=2^{4}-1=15. \end{array}$$

The first four features are the original features from the base dataset (*P*, *A*, *L*, *R*), while the remaining features are generated from the average values of the base dataset features.

To evaluate the quality of the new features, we performed *t*-tests for each feature in *S* ^*T*^. The hypothesis H_0_ states that “the FA value of a normal slice is the same as the FA value of an injured slice.” The *p* value of *t*-test for each feature is presented in Table 2. All the *p* values are lower than 0.05, which indicates that hypothesis H_0_ should be rejected. In other words, the FA value of a normal slice is significantly different from the FA value of a patient slice in every feature. It also implies that the 15 given features are sufficient for classification analysis.

The extended dataset has 15 features to which we can apply the feature selection scheme to improve classification accuracy. Various feature selection schemes, with a varying number of features needed for optimal classification accuracy, are available for use. In our experiment, we tested four feature selection algorithms: FSDD, ReliefF, CBFS, and RFS. Each feature selection algorithm has a feature evaluation function. After evaluating each feature in the dataset, we chose *n* features that had the best score. The next step was classification analysis using these *n* features.

### 3.3. Classification Analysis

To acquire the most accurate results from the extended dataset, we evaluated the quality of each feature using various feature selection algorithms. We then used a set of high-qualified features for classification analysis. The KNN and SVM supervised learning algorithms were used as classifiers for the target dataset.

## 4. Results and Discussion

### 4.1. Classification of Patients and Normal, Healthy Individuals

To classify patients and normal, healthy controls, we modified the extended dataset because a single individual has 14-15 slice data points and direct comparison with other individuals is difficult. The average FA values of entire slices were calculated for each individual and a modified dataset was constructed. We used the KNN classifier with *k* = 1 and performed the Leave-One-Out Cross-Validation (LOOCV) test [21] because the data sample size of the modified dataset was small.  Table 3 presents the classification results. These results show 100% classification accuracy, indicating that the proposed dataset is useful in the classification of normal, healthy individuals and SCI patients.

### 4.2. Classification of Normal and Patient Slices

**Figure 6:**
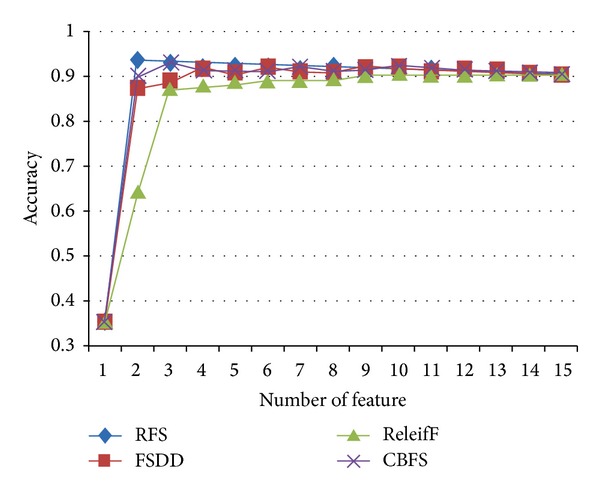
The graph of the accuracy by each feature using KNN algorithms with *k* = 5.

The test outlined in this section was performed to predict which part of the SC is injured. This test is more difficult than the diagnosis of injured patients in Section 4.1. To classify normal individuals and patients' slices, we used an entire extended dataset and the KNN classifier. We tested the influence of feature selection using RFS, FSDD, ReliefF, and CBFS. From the experimental results, we found that RFS selects the best features that produce high classification accuracy compared with the other feature selection algorithms.  Figure 6 shows the classification accuracy for the entire feature list. The classification accuracy is obtained by applying the four feature selection algorithms with KNN and SVM classifications. In KNN, the choice of *k* affects the performance of the algorithm. We set *k* = 5 for KNN and used threefold cross-validation to avoid overfitting problems. In Figure 6, the number of features refers to the number of selected features for the classification test. For example, if the number of features is six, then the best six features evaluated by the four feature selection algorithms are used for the classification test. It can be seen that two features from the RFS algorithm give a superior accuracy of 93.8%. The accuracy measurements of FSDD, ReliefF, and CBFS (87.7%, 64.2%, and 90.1%, resp.) are not competitive with that of RFS. From the results depicted in Figure 6, we know that two or three is the optimal number of features for achieving the best classification accuracy.  Table 4 shows the features and *R*-values for RFS. The *R*-value scores are ranked in ascending order; a lower *R*-value score indicates better quality features.  Table 5 presents a comparison of the best classes among the base dataset, extended dataset, and the feature selection dataset. We adopted RFS for feature selection.  Table 6 describes the experiment variables presented in Table 5. The applied dataset produced better classification accuracy than the base and extended datasets, while the proposed modified dataset that was collected by the RFS algorithm proved to be better than the original base dataset (Table 5). These results indicate that both the FA value and the SC region are important factors for SCI prediction; the latter is one of the main aspects influencing the classification result.

Table 7 shows the sensitivity and specificity analysis results, which is another way to evaluate classification results apart from accuracy. The results of a classification are generally assessed using the following measures: True Positive (TP), True Negative (TN), False Positive (FP), and False Negative (FN).

Accuracy (i.e., the proportion of correctly classified samples) is defined by

(5) $$\begin{array}{c} \text{Accuracy}=\frac{\left(\text{TP}+\text{TN}\right)}{\left(\text{TP}+\text{TN}+\text{FP}+\text{FN}\right)}. \end{array}$$

Sensitivity (i.e., the proportion of correctly classified positive samples) is defined by

(6) $$\begin{array}{c} \text{Sensitivity}=\frac{\text{TP}}{\left(\text{TP}+\text{FN}\right)}. \end{array}$$

Specificity (i.e., the proportion of correctly classified negative samples) is defined by

(7) $$\begin{array}{c} \text{Specificity}=\frac{\text{TN}}{\left(\text{TN}+\text{FP}\right)}. \end{array}$$

In Table 7, the normal case specificity is 0.912 and the abnormal case sensitivity is 0.952. These results indicate that the proposed dataset has good predictive power for both normal and injured slices.

## 5. Conclusion

Diffusion Tensor Imaging (DTI) is generally used in the analysis of the brain and brain injuries. In recent years, this technique has been applied to Spinal Cord Injury (SCI) analysis. In this paper, we proposed a machine learning scheme for the diagnosis of SCI. We outlined a method of building datasets for diagnosis by observing four FA values from DTI slice images of patients and normal controls. Extension of the base dataset to a 15-feature dataset is one of the main contributions made in this study. Feature selection with the extended dataset resulted in improved prediction accuracy. A limitation of the study is that we did not acquire a large amount of SC data due to legal limitations for medical data. Nevertheless, we believe that if we gather more data, we can improve our dataset and prediction accuracy. Our dataset is designed for specific cases of SCI in the C4–C6 area. A different area of injury will require a different kind of dataset. However, our approach can be used to build a suitable dataset for any such area. The ultimate goal of our study is to predict injured SC slices using a predefined training dataset. In the future, we plan to extend the current dataset with additional information so as to improve the prediction accuracy.

## Figures and Tables

**Algorithm 1:**
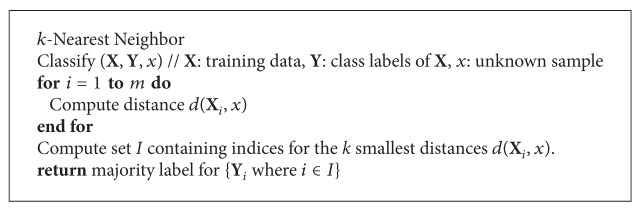
Pseudocode for KNN classification.

**Table 1:** Example of a base dataset.

Class label	Posterior	Anterior	Left	Right
0	0.37388	0.331959	0.401053	0.511554
0	0.071062	0.336189	0.101144	0.434287
0	0.154871	0.532321	0.338796	0.30359
0	0.51665	0.710171	0.475486	0.482532
*⋮*	⋮	⋮	⋮	⋮
1	0.728015	0.553007	0.673909	0.484319
1	0.569538	0.538663	0.715141	0.427902
1	0.720117	0.527224	0.642175	0.535785
1	0.617641	0.482621	0.573918	0.424463
*⋮*	⋮	⋮	⋮	⋮

**Table 2:** Results of *t*-tests for each feature in the target dataset.

	Feature	*p* value = *P* (*T* ≤ *t*)	AVG (patient)	AVG (normal)
1	*P*	7.69∗10^−9^	0.427	0.535
2	*A*	8.05∗10^−31^	0.451	0.684
3	*L*	1.89∗10^−20^	0.475	0.645
4	*R*	2.39∗10^−26^	0.419	0.610
5	(*P*, *A*)	3.03∗10^−23^	0.439	0.609
6	(*P*, *L*)	8.37∗10^−18^	0.451	0.590
7	(*P*, *R*)	5.61∗10^−19^	0.423	0.573
8	(*A*, *L*)	8.99∗10^−31^	0.463	0.665
9	(*A*, *R*)	2.74∗10^−33^	0.435	0.647
10	(*L*, *R*)	4.35∗10^−31^	0.447	0.628
11	(*P*, *A*, *L*)	4.95∗10^−26^	0.451	0.621
12	(*A*, *L*, *R*)	2.05∗10^−34^	0.448	0.647
13	(*P*, *A*, *R*)	8.57∗10^−27^	0.432	0.610
14	(*P*, *L*, *R*)	3.3∗10^−24^	0.440	0.597
15	(*P*, *A*, *L*, *R*)	4.62∗10^−29^	0.443	0.619

H_0_: the FA value of a normal slice is the same as the FA value of a patient slice; AVG: average of FA value.

**Table 3:** Classification results of patients and normal individuals.

	Diagnosis result	1-NN result using LOOCV
Person 1	Patient	Patient
Person 2	Patient	Patient
Person 3	Patient	Patient
Person 4	Patient	Patient
Person 5	Patient	Patient
Person 6	Patient	Patient
Person 7	Patient	Patient
Person 8	Patient	Patient
Person 9	Patient	Patient
Person 10	Normal	Normal
Person 11	Normal	Normal
Person 12	Normal	Normal
Person 13	Normal	Normal
Person 14	Normal	Normal

**Table 4:** Results of feature evaluation using the RFS algorithms.

Ranking	Index of feature	Feature	*R*-value
1	11	(*A*, *L*, *R*)	0.060976
2	8	(*A*, *R*)	0.067073
3	9	(*L*, *R*)	0.085366
4	14	(*P*, *A*, *L*, *R*)	0.085366
5	13	(*P*, *L*, *R*)	0.085366
6	12	(*P*, *A*, *R*)	0.085366
7	7	(*A*, *L*)	0.091463
8	3	*R*	0.091463
9	1	*A*	0.097561
10	2	*L*	0.103659
11	10	(*P*, *A*, *L*)	0.109756
12	4	(*P*, *A*)	0.115854
13	6	(*P*, *R*)	0.146341
14	5	(*P*, *L*)	0.146341
15	0	*P*	0.158537

**Table 5:** The best accuracy of the base dataset, extended dataset, and feature selection applied dataset using KNN and SVM.

	Base dataset (4 features)	Extended dataset (15 features)	FS applied dataset (*n* feature)
KNN	0.901	0.914	0.938 (*n* = 2)
SVM	0.932	0.927	0.933 (*n* = 8)

**Table 6:** The experimental variables for the best accuracies in Table 3.

	Base dataset (user-defined value)	Modified dataset (user-defined value)	Best FS dataset (FS/no. of feature/user-defined value)
KNN	*k* = 15	*k* = 3	RFS/2/*k* = 5
SVM	Radial basis function	Linear	CBFS (FSDD)/8/linear kernel

**Table 7:** Accuracy, sensitivity, and specificity.

	Prediction	
	Injured	Normal
Fact		
Injured	100 (TP)	5 (FN)
Normal	5 (FP)	52 (TN)

Sensitivity = 0.952, specificity = 0.912, accuracy = 0.938.

## References

[1] The American Spinal Injury Association (1982). *Standard for Neurological Classification of Spinal Injured Patients*.

[2] The American Spinal Injury Association/International Medical Society of Paraplegia (1996). *International Standards for Neurological and Functional Classification of Spinal Cord Injury Patients*.

[3] The American Spinal Injury Association/International Medical Society of Paraplegia (2000). *International Standards for Neurological and Functional Classification of Spinal Cord Injury Patients*.

[4] Steeves JD, Lammertse D, Curt A (2007). Guidelines for the conduct of clinical trials for spinal cord injury (SCI) as developed by the ICCP panel: clinical trial outcome measures. *Spinal Cord*.

[5] Miyanji F, Furlan JC, Aarabi B, Arnold PM, Fehlings MG (2007). Acute cervical traumatic spinal cord injury: MR imaging findings correlated with neurologic outcome. Prospective study with 100 consecutive patients. *Radiology*.

[6] Lin T, Li R, Tang X, Dy JG, Jiang SB (2009). Markerless gating for lung cancer radiotherapy based on machine learning techniques. *Physics in Medicine and Biology*.

[7] Norman KA, Polyn SM, Detre GJ, Haxby JV (2006). Beyond mind-reading: multi-voxel pattern analysis of fMRI data. *Trends in Cognitive Sciences*.

[8] Athitsos V, Alon J, Sclaroff S Efficient nearest neighbor classification using a cascade of approximate similarity measures.

[9] Chang C-C, Lin C-J (2011). LIBSVM: a library for support vector machines. *ACM Transactions on Intelligent Systems and Technology*.

[10] Schölkopf B, Smola AJ (2002). *Learning With Kernels: Support Vector Machines, Regularization, Optimization, and Beyond*.

[11] Zare H FeaLect: Feature seLection by computing statistical scores. http://cran.rakanu.com.

[12] Liang J, Yang S, Winstanley A (2008). Invariant optimal feature selection: a distance discriminant and feature ranking based solution. *Pattern Recognition*.

[13] Robnik-Šikonja M, Kononenko I (2003). Theoretical and empirical analysis of reliefF and RReliefF. *Machine Learning*.

[14] Seo M, Oh S (2012). High performance feature selection algorithm based on feature clearness (CBFS). *PLoS ONE*.

[15] Lee J, Nomin B, Oh S (2012). RFS :efficient feature selection method based on R-value. *Computers in Biology and Medicine*.

[16] Oh S (2011). A new dataset evaluation method based on category overlap. *Computers in Biology and Medicine*.

[17] American Spinal Injury Association/International Medical Society of Paraplegia (2003). *International Standards for Neurological and Functional Classification of Spinal Cord Injury Patients*.

[18] Dirk-Jan K http://www.mathworks.com/matlabcentral/fileexchange/21130-dti-and-fiber-tracking.

[19] Jimmy S http://www.mathworks.com/matlabcentral/fileexchange/8797.

[20] Cohen-Adad J, Leblond H, Delivet-Mongrain H, Martinez M, Benali H, Rossignol S (2011). Wallerian degeneration after spinal cord lesions in cats detected with diffusion tensor imaging. *NeuroImage*.

[21] Arlot S, Celisse A (2010). A survey of cross-validation procedures for model selection. *Statistics Surveys*.

